# The impact of problem domain on Bayesian inferences: A systematic investigation

**DOI:** 10.3758/s13421-023-01497-1

**Published:** 2024-01-10

**Authors:** Stefania Pighin, Flavia Filimon, Katya Tentori

**Affiliations:** 1 Center for Mind/Brain Sciences (CIMeC), University of Trento, Corso Bettini n. 31, 38068 Rovereto, TN Italy; 2 Center for Medical Sciences (CISMed), University of Trento, Trento, Italy

**Keywords:** Bayesian inference, Bayesian reasoning, Posterior probability, Natural frequency format, Bayesian word problems

## Abstract

Sparse (and occasionally contradictory) evidence exists regarding the impact of domain on probabilistic updating, some of which suggests that Bayesian word problems with medical content may be especially challenging. The present research aims to address this gap in knowledge through three pre-registered online studies, which involved a total of 2,238 participants. Bayesian word problems were related to one of three domains: medical, daily-life, and abstract. In the first two cases, problems presented realistic content and plausible numerical information, while in the latter, problems contained explicitly imaginary elements. Problems across domains were matched in terms of all relevant statistical values and, as much as possible, wording. Studies 1 and 2 utilized the same set of problems, but different response elicitation methods (i.e., an open-ended and a multiple-choice question, respectively). Study 3 involved a larger number of participants per condition and a smaller set of problems to more thoroughly investigate the magnitude of differences between the domains. There was a generally low rate of correct responses (17.2%, 17.4%, and 14.3% in Studies 1, 2, and 3, respectively), consistent with accuracy levels commonly observed in the literature for this specific task with online samples. Nonetheless, a small but significant difference between domains was observed: participants’ accuracy did not differ between medical and daily-life problems, while it was significantly higher in corresponding abstract problems. These results suggest that medical problems are not inherently more difficult to solve, but rather that performance is improved with abstract problems for which participants cannot draw from their background knowledge.

## Introduction

The ability to update probabilities based on new evidence is a fundamental aspect of human cognition, allowing us to learn, solve problems, and make rational decisions (Chater & Oaksford, 2008; Sutton & Barto, 2018; Tversky & Kahneman, 1974). However, research has repeatedly shown that even highly educated individuals struggle with this form of reasoning when assessed by *Bayesian word problems* (also known as *textbook problems*; Barbey & Sloman, 2007; Bar-Hillel, 1980; Kahneman & Tversky, 1973). Typically, such problems provide explicit numerical information concerning a hypothesis (e.g., the prevalence of a disease) together with the relation between a piece of evidence (e.g., a diagnostic test result) and the hypothesis under consideration (i.e., the true and the false positive rates of the test). Individuals are then asked to calculate the posterior probability of the hypothesis based on the given evidence (i.e., the positive predictive value of the test).

The domain of word problems is inconsequential from a Bayesian perspective, as the only relevant information is the likelihood of the evidence under each alternative hypothesis (i.e., to have vs. not to have the disease) and the prior probabilities of the hypothesis at issue (i.e., to have the disease). In line with this, a number of experimental studies have not reported significant differences in performance on Bayesian word problems across different domains (e.g., Chapman & Liu, 2009; Micallef, Dragicevic, & Fekete, 2012; Pighin, Tentori, & Girotto, 2017). Nevertheless, the assumption that the domain does not matter, as long as the relevant probability values are communicated effectively, remains controversial, as the evidence is mixed (e.g., Binder, Krauss, & Bruckmaier, 2015; Bruckmaier, Binder, Krauss, & Kufner, 2019; Siegrist & Keller, 2011; Sirota, Juanchich, & Hagmayer, 2014). Such mixed results make it challenging to draw firm conclusions about the impact of domain on solving Bayesian word problems. This is especially so considering that previous studies have varied the domain of the problems along with changes to the probability values, the language used to convey the relevant information, as well as the length and the complexity of the text. To the best of our knowledge, Siegrist and Keller’s study (2011; Experiment 4) appears to be the only one to have presented participants with medical and non-medical problems in a between-subject design, at least by matching numerical values. Their results indicated that participants were more likely to solve a Bayesian problem correctly when it was in a non-medical domain compared to a medical one. In the authors’ interpretation, the greater difficulty with problems in the medical domain stemmed from the technical medical terminology employed, which could be challenging to grasp for the general population. Alternatively, they suggested that non-medical problems may have been perceived as less daunting and more easily understood by non-experts too. A different explanation ascribes the difficulty of medical problems to the extremely low base-rates they typically use, which would make the correct answer seem counterintuitive to participants (Binder, Krauss, & Bruckmaier, 2015). It should be noted, however, that this account cannot be applied to Siegrist and Keller’s findings since, as mentioned above, they matched the values, including that referring to the base rate, between scenarios. Finally, an alternative rationale for a possible greater difficulty in medical problems pertains to participants’ misperception of medical tests as being virtually infallible, despite being provided with information to the contrary (Hammerton, 1973). This would align with the well-documented tendency to overestimate the sensitivity of diagnostic and screening tests (Lyman & Balducci, 1993; Pighin & Tentori, 2021; Steurer et al., 2002), suggesting that individuals may over-rely on them and underestimate or dismiss the possibility of false results.

Overall, due to the lack of systematicity in previous studies, it is still unclear whether Bayesian inferences are more challenging to handle in the medical domain and, if so, what the cause of this might be. Indeed, while some studies (Hafenbrädl & Hoffrage, 2015; Johnson & Tubau, 2015) have recognized the importance of examining the potential impact of specific characteristics of textbook problems on participants’ cognitive processes (e.g., whether the hypothesis under consideration can be considered unusual vs. a norm or whether the problem features high vs. low stakes), an investigation into the influence of problem domain has yet to be undertaken. Our study aimed to fill this gap by exploring the effects of three types of domains on Bayesian reasoning problems.

## The present research

In three online pre-registered studies, we asked participants to solve isomorphic Bayesian word problems, presenting numerical information in a natural frequency format. This format was chosen in order to reduce computational complexity and improve reasoning accuracy (Gigerenzer & Hoffrage, 1995). Our exploratory research focused on the following two aspects.

Firstly, we investigated whether, ceteris paribus (i.e., holding all relevant probability values constant and minimizing linguistic differences between problems), accuracy was affected by the domain of the problem, with medical problems leading to lower accuracy compared to non-medical problems. Three medical problems were selected, varying both in terms of content (specifically, celiac disease, Down syndrome, and osteoarthritis) and the relevant probabilistic information (i.e., base rates, true positives, and false positives). Given that non-medical problems can encompass a wide range of domains, we sought to employ in our investigation both real-life non-medical problems, for which individuals may possess some background knowledge (hereafter referred to as “daily-life problems”), and abstract problems, for which no prior knowledge is possible since they included explicitly imaginary elements (for the complete list of verbatim problems used, please refer to the Appendix). Daily-life problems (i.e., the gold coins, organic apples, and alkalinity problems) were chosen from a bigger pool of potential real-life problems that were specifically generated for this research. The selection of these three problems was based on their better alignment, in terms of values plausibility and word count, with the three medical problems. Similarly, abstract problems were aligned to the daily-life and medical problems with regard to word count and fully matched with them in terms of values. They referred to a hypothetical planet inhabited by flying creatures, and their imaginary content was expected to neutralize any potential impact of prior knowledge.

Secondly, we examined whether accuracy in Bayesian inferences was influenced by the type of evidence at stake. More specifically, we investigated if accuracy differed when, as in classical medical problems, evidence concerned the outcome of a test (e.g., receiving a positive prenatal screening result) or, instead, a property, feature, or action (e.g., attending a genetic counseling support group) that were probabilistically equivalent in their association with the same hypothesis (e.g., carrying a child with Down syndrome). Indeed, even if the probability associations under consideration are exactly the same, these problems may be perceived differently by human reasoners. Specifically, in the case of tests, two of the four possible outcomes of the combination between evidence and hypothesis represent errors (i.e., the false negatives and the false positives). Such errors are assumed to be randomly distributed, meaning that although their overall proportions are expected to remain relatively constant across repeated tests, this won’t necessarily hold for individual cases (e.g., a specific false negative result can become a true negative in a subsequent round of the same test). In contrast, when associations between properties are involved, it makes no sense to talk about errors, and single cases are completely determined (e.g., a particular woman, who is carrying a child with Down syndrome, is or is not attending a genetic counseling support group, and repeating the sampling will not alter this fact). Since variables such as ambiguity are known to lower individuals’ confidence in their judgements, by influencing, for example, willingness to bet (Ellsberg, 1961; Heath & Tversky, 1991) and assessment of evidential impact (Tentori, Crupi, & Osherson, 2007), it is reasonable to assume that they may affect probability updating as well. Furthermore, the manipulation of the type of evidence allowed us to test the above-mentioned explanation that ascribes the difficulties in the medical domain to a common misperception of medical tests as infallible (Hammerton, 1973). If this explanation were correct, we would expect lower accuracy in Bayesian word problems whose evidence pertains to the outcome of a test rather than a property or feature probabilistically associated with the hypothesis at issue, at least in the medical domain.

## Study 1

### Method

#### Participants

The minimum sample size needed for Study 1 was computed by performing an a priori power analysis using G*Power 3.1 (Faul et al., 2009), which indicated a minimum of 39 participants per condition to detect a small/medium effect size of 0.20, assuming *α* = .05 and 1 – *β* = .90. The survey was kept active until at least 40 participants completed the task for each of the 18 conditions. Accordingly, we recruited 762 UK residents (*M*_age_ = 42 years, *SD* = 13.4; 315 men, 446 women, one participant preferred not to declare their gender) using the Prolific platform. Most of them had an undergraduate (39.2%) or a graduate degree (17.2%), some had at least some college/university (24.1%), and the remaining participants were educated up to the level of high school diploma (19.5%). There were no time limits for task completion, and participants received compensation of 0.63 British pounds (ensuring an hourly payment of £7.50, in accordance with Prolific guidelines) for their participation.

#### Materials and design

The pre-registered protocol of Study 1 can be found at https://osf.io/2da5k. Study 1 employed a full between-subject design, in which two independent variables were manipulated: the domain of the problem (medical, daily-life, vs. abstract) and the type of evidence (testing vs. non-testing). To increase the generalizability of our findings, the problems were generated by using three different combinations of prior, true positives, and false positives values (see Table 1), for a total of 18 problems (i.e., 3 domains × 2 types of evidence × 3 value combinations, see Appendix). Such combinations of values were chosen to ensure that the numerical information provided in all problems was plausible. This means that not only were all the values pertaining to the prevalence of the conditions, test characteristics, and associations in the medical problems matched to the actual ones, but this was also the case for the values presented in corresponding daily-life problems. The content of the abstract problem was kept constant and its values were matched to those of the medical and daily-life problems.

**Table 1:** The three combinations of priors and test characteristic values used in the present research. Studies 1 and 2 employed all three value combinations, while Study 3 employed only value combinations 2 and 3

Value combinations	Base rate	True positives	False positives
1	7 out of 1,000	6 out of 7	50 out of 993
2	10 out of 1,000	8 out of 10	79 out of 990
3	13 out of 100	9 out of 13	20 out of 87

The main dependent variable was the accuracy of participants’ responses to an open-ended probability question framed in a natural frequencies format (see Appendix), which resembled the standard question employed in previous studies (e.g., Gigerenzer & Hoffrage, 1995; Pighin, Gonzalez, Savadori, & Girotto, 2016). Only responses that were equivalent to the correct Bayesian answer were considered accurate. Answers other than the correct solution were classified into one of the following categories, which summarize various non-Bayesian strategies that have been reported with adult participants (e.g., Gigerenzer & Hoffrage, 1995; Pighin, Girotto, & Tentori, 2017):

- “Sensitivity,” which represents how often the evidence (*E*) occurs when the hypothesis (*H*) is true (i.e., *p*(*E*|*H*), for example “6 out of 7” in the value combination 1);
- “Base-rate only,” which only considers the prior probability, while the evidence is disregarded (i.e., *p*(*H*), for example “7 out of 1,000” in the value combination 1);
- “Evidence-only,” which focuses on the occurrence of the evidence among all cases (i.e., *p*(*E*), for example “56 out of 1,000” in the value combination 1);
- “Joint occurrence,” which indicates how often both the evidence and the hypothesis occur among all cases (i.e., *p*(*H*&*E*), for example, “6 out of 1,000” in the value combination 1).

We also added a fifth category, named “Specificity,” which conveys how often the evidence does not occur when the hypothesis is false (i.e., *p*(not-*E*|not-*H*), for example “943 out of 993” in the value combination 1). Incorrect answers that escaped the above categories were classified as “Other” (e.g., responses like “10 out of 517”, “20 out of 1,000”, or “50 out of 993” in the value combination 1).

Finally, a multiple-choice question was included at the end of the task in order to check whether participants considered the probability values used in the medical and daily-life problems to be believable. The question read as follows:

“The numerical values that I was provided with in the above problem are: …” and participants had to complete the sentence above by choosing one of following four options:^1^

- “believable to me (they are aligned with my knowledge about this content)”;
- “believable to me (I do not have any knowledge about this content)”;
- “partially believable to me (they are partially aligned with my knowledge about this content)”;
- “unbelievable to me (they are not aligned with my knowledge about this content)”.

It should be noted that, since the abstract problem involved an imaginary scenario, it could not be evaluated in these terms.

### Results

The majority of participants indicated that the numerical values provided in the problems were believable to them (either because the values aligned with their knowledge about the problem content, 8.9%, or because they had no knowledge about it, 68%); 15.4% of participants indicated that the values they were presented with were at least partially believable and aligned with their knowledge; while only 7.7% of participants indicated that the values were unbelievable to them and not aligned with their knowledge. Importantly, the distribution of participants’ answers did not differ significantly between medical and daily-life problems (*χ*^2^(3, *N* = 506) = 5.13, *p* = .163, *BF*_10_ = .166^2^), even when domains were considered separately within each type of evidence and value combination (all *p*s > .05).

Accuracy rates and the distribution of incorrect responses in the 18 problems of Study 1 are reported in Tables 2 and 3, respectively. Overall, participants’ accuracy was low (17.2%), and did not differ among the 18 problems *χ*^2^(17, *N* = 762) = 16.27, *p* = .505, *BF*_10_ < 0.001. However, the results of a logistic regression analysis^3^ on accuracy rate, which included domain, type of evidence, and value combination as categorical predictors, showed that domain was a significant predictor of participants’ accuracy (*χ*^*2*^(2) = 7.39, *p* = .007). Specifically, a comparison among the three domains (with Bonferroni correction) indicated that participants were less accurate in the medical domain than in the abstract one (*OR* = .520, 95% CI, .321–.840), with no difference between the medical and the daily-life problems or between the daily-life and the abstract problems (both *p*s > .05). The type of evidence and the value combination were not significant predictors of participants’ accuracy (all *p*s > .05).

**Table 2:** Accuracy rates (i.e., percentages of Bayesian responses) for the 18 experimental conditions of Studies 1 and 2. Refer to Table 1 for an explanation of values 1, 2, and 3

Domain	Type of evidence	Value	Study 1		Study 2	
			N	Accuracy %	N	Accuracy %
Medical	Testing	1	41	9.8	43	14.0
		2	41	14.6	42	9.5
		3	42	11.9	41	7.3
		Overall	124	12.1	126	10.3
	Non-testing	1	43	11.6	40	20.0
		2	41	22.0	41	19.5
		3	42	4.8	41	4.9
		Overall	126	12.7	122	14.8
	Overall		250	12.4	248	12.5
Daily-life	Testing	1	43	11.6	41	19.5
		2	46	17.4	41	19.5
		3	40	20.0	45	17.8
		Overall	129	16.3	127	18.9
	Non-testing	1	40	20.0	40	17.5
		2	46	19.6	40	22.5
		3	41	17.1	44	15.9
		Overall	127	18.9	124	18.5
	Overall		256	17.6	251	18.7
Abstract	Testing	1	40	17.5	40	22.5
		2	47	17.0	40	22.5
		3	42	28.6	42	21.4
		Overall	129	20.9	122	22.1
	Non-testing	1	40	22.5	41	17.1
		2	47	21.3	42	23.8
		3	40	22.5	43	18.6
		Overall	127	22.0	126	19.8
	Overall		256	21.5	248	21.0
Overall			762	17.2	747	17.4

**Table 3:** Percentages of non-Bayesian responses falling into the six incorrect categories in Studies 1 and 2. Refer to Table 1 for an explanation of values 1, 2, and 3

Domain	Type of evidence	Value combination	Study 1							Study 2					
			N	Non-Bayesian responses						N	Non-Bayesian responses				
				Sensitivity	Base rate	Evidence Only	Joint occurrence	Specificity	Other		Sensitivity	Base rate	Evidence only	Joint occurrence	Specificity
Medical	Testing	1	41	9.8	17.1	12.2	7.3	0.0	43.9	43	16.3	20.9	14.0	32.6	2.3
		2	41	7.3	17.1	7.3	14.6	0.0	39.0	42	14.3	32.7	11.9	28.6	0.0
		3	42	2.4	21.4	21.4	14.3	0.0	28.6	41	7.3	43.9	14.6	19.5	7.3
		Overall	124	6.5	18.5	13.7	12.1	0.0	37.1	126	12.7	33.3	13.5	27.0	3.2
	Non-testing	1	43	14.0	14.0	9.3	11.6	0.0	39.5	40	7.5	35.0	20.0	17.5	0.0
		2	41	4.9	19.5	2.4	7.3	0.0	43.9	41	9.8	22.0	14.6	26.8	7.3
		3	42	11.9	31.0	19.0	9.5	0.0	23.8	41	7.3	43.9	29.3	12.2	2.4
		Overall	126	10.3	21.4	10.3	9.5	0.0	35.7	122	8.2	33.6	21.3	18.9	3.3
	Overall		250	8.4	20.0	12.0	10.8	0.0	36.4	248	10.5	33.5	17.3	23.0	3.2
Daily-life	Testing	1	43	0.0	23.3	18.6	2.3	0.0	44.2	41	2.4	31.7	29.3	14.6	2.4
		2	46	13.0	32.6	4.3	8.7	0.0	23.9	41	12.2	34.1	7.3	22.0	4.9
		3	40	5.0	0.0	42.5	5.0	0.0	27.5	45	11.1	35.6	24.4	8.9	2.2
		Overall	129	6.2	19.4	20.9	5.4	0.0	31.8	127	8.7	33.9	20.5	15.0	3.1
	Non-testing	1	40	2.5	27.5	2.5	10.0	0.0	37.5	40	10.0	27.5	15.0	27.5	2.5
		2	46	8.7	19.6	4.3	6.5	0.0	41.3	40	7.5	22.5	15.0	22.5	10.0
		3	41	19.5	9.8	2.4	14.6	4.9	31.7	44	20.5	29.5	20.5	6.8	6.8
		Overall	127	10.2	18.9	3.1	10.2	1.6	37.0	124	12.9	26.6	16.9	18.5	6.5
	Overall		256	8.2	19.1	12.1	7.8	0.8	34.4	251	10.8	30.3	18.7	16.7	4.8
Abstract	Testing	1	40	7.5	15.0	5.0	15.0	0.0	40.0	40	10.0	27.5	20.0	17.5	2.5
		2	47	17.0	21.3	6.4	2.1	0.0	36.2	40	17.5	20.0	22.5	15.0	2.5
		3	42	7.1	23.8	11.9	11.9	0.0	16.7	42	14.3	21.4	16.7	23.8	2.4
		Overall	129	10.9	20.2	7.8	9.3	0.0	31.0	122	13.9	23.0	19.7	18.9	2.5
	Non-testing	1	40	10.0	7.5	5.0	15.0	0.0	40.0	41	9.8	22.0	9.8	39.0	2.4
		2	47	14.9	8.5	4.3	14.9	0.0	36.2	42	16.7	16.7	14.3	23.8	4.8
		3	40	7.5	15.0	17.5	15.0	0.0	22.5	43	20.9	16.3	11.6	23.3	9.3
		Overall	127	11.0	10.2	8.7	15.0	0.0	33.1	126	15.9	18.3	11.9	28.6	5.6
	Overall		256	10.9	15.2	8.2	12.1	0.0	32.0	248	14.9	20.6	15.7	23.8	4.0
Overall			762	9.2	18.1	10.8	10.2	0.3	34.3	747	12.0	28.1	17.3	21.2	4.0

The majority of participants’ incorrect responses fell into the “Other” category (34.3% across conditions), suggesting that they were mainly random (e.g., summing or subtracting values arbitrarily picked from the problem text). Among the remaining categories, the strategy of relying only on the prior probability (“Base-rate only”) was the most systematic error (19.1%; for a similar result see Pighin, Girotto, & Tentori, 2017). Out of the 762 judgments, only two (0.03%) errors fell into the “Specificity” category, indicating an extremely low incidence of such error. The other three errors (“Sensitivity,” “Evidence only,” and “Joint occurrence” categories) occurred at roughly similar rates (around 10% each, see Table 3). Approximately the same pattern of errors was observed within each domain, within each type of evidence, and within each value combination (with one single exception in the problems with value combination 3, where the “Base-rate only” was the second most common error, 20.6%, after the “Evidence only” one, 23.0%).

Consistent with Siegrist and Keller (2011), participants in our study showed the lowest accuracy when making judgments about medical problems. However, we found that participants’ judgments were significantly less accurate in the medical domain when compared to the abstract domain, while no significant difference was observed between the daily-life domain and the other two domains. The distribution of errors was largely consistent across conditions and independent from domain and type of evidence. However, it is important to note that, despite aligning with existing literature, overall accuracy rates were low (for further discussion on the low accuracy obtained, see the following section) and this could have limited the possibility to observe specific differences between conditions.

## Study 2

In Study 1, we evaluated participants’ accuracy in responding to an open-ended probability question, which was consistent with the methodology used in the majority of previous studies. However, due to the low rate of correct responses and a high rate of unclassifiable errors, we conducted a second study to investigate whether participants could at least recognize the correct response when presented as one of several response options (for a similar rationale of using a multiple-choice question to simplify Bayesian inferences, see also McNair & Feeney, 2014; Msaouel et al., 2015). In order to do so, in Study 2, we presented participants with the same Bayesian problems used in Study 1, along with a list of possible response options (see below for more details on these options).

### Method

#### Participants

As in Study 1, the survey was kept active until at least 40 participants completed the task for each of the 18 conditions. Accordingly, we recruited a new sample of 747 UK residents (*M*_age_ = 42 years, *SD* = 13.3; 332 men, 414 women) using the Prolific platform. Participants’ education level was comparable to that of Study 1: most participants had an undergraduate (40.6%) or a graduate degree (17.0%), some had at least some college/university (24.9%), and the remaining participants completed up to high school diploma (17.5%). Participants received the same compensation as in Study 1.

#### Materials and design

The pre-registered protocol of Study 2 can be found at https://osf.io/ckwde. Study 2 employed the same full between-subject design and materials (see Appendix) used in Study 1. Participants, however, had to answer a multiple-choice question (instead of an open-ended question). The question read exactly as in Study 1, but six alternative response options were provided, in a random order: the correct answer along with the answers that correspond to the five error categories of Study 1 (i.e., “Sensitivity,” “Base-rate only,” “Evidence only,” “Joint occurrence,” and “Specificity”). At the end of the task, participants were posed the same multiple-choice question used in Study 1 to check whether the probability values included in the medical and daily-life problems appeared believable to them.

#### Results

Similar to Study 1, the majority of participants indicated that the numerical values provided in the problems were believable to them (9.1% because the values were aligned with their knowledge about the problem content, and 69.5% because they had no knowledge about the content at issue). About 17% of participants found the values to be partially believable to them and consistent with their knowledge, while only 4.4% of participants found the values to be unbelievable to them and inconsistent with their knowledge. In Study 2, the distribution of participants’ answers differed significantly between medical and daily-life problems: a higher rate of participants found the values to be believable and consistent with their knowledge in the medical problems compared to the daily-life one (12.5% vs. 5.6%, respectively; (*χ*^2^(3, *N* = 490) = 8.47, *p* = .037, *BF*_10_ = 0.517). Importantly, however, the proportion of participants who found the values to be at least partially believable and consistent with their knowledge was approximately the same in the two domain conditions (i.e., 16.1% and 17.9% in the medical and in the daily-life domains, respectively). The same held for the proportion of participants who found the values to be unbelievable and inconsistent with their knowledge (i.e., 5.2% and 3.6% in the medical and in the daily-life domain, respectively).

Overall, the accuracy rate was low (17.4%) and fully comparable with that obtained in Study 1. Similarly, no significant differences were observed among the 18 problems, *χ*^2^(17, *N* = 747) = 14.07, *p* = .662, *BF*_10_ < 0.001. As in Study 1, the results of a logistic regression analysis on accuracy rate, which included domain, type of evidence, and value combination as categorical predictors, showed that participants were less accurate in the medical domain than in the abstract one (*OR* = .536, 95% CI, .330–.871; *p* = .012), while no other difference was observed (i.e., between the medical and the daily-life problems or between the daily-life and the abstract problems, all *p*s > .05).^4^ As in Study 1, the type of evidence and the value combination did not significantly predict participants’ accuracy rate (all *p*s > .05).

The analysis of participants’ non-Bayesian responses confirmed that the most common error was represented by the choice of the “Base-rate only” option (28.1% across conditions). The second and third most frequent errors were given by the selection of the “Joint occurrence” (21.2%) and the “Evidence only” (17.3%) options, while participants were less inclined to opt for responses corresponding to the “Sensitivity” (12%) or “Specificity” (4%) categories. No significant differences were observed in the distribution of errors among domains, among types of evidence, or among value combinations (all *p*s > .05).

Notably, participants’ performance in Study 2 did not improve with the use of multiple-choice questions that only required them to identify the correct response: across all problems, the accuracy rate remained low and fully comparable to that observed in Study 1, when an open question was employed. The results of Study 2 were also consistent with those of Study 1 with regard to participants’ lower accuracy with Bayesian problems in the medical domain compared to the abstract one. Yet again, the accuracy rate on daily-life problems fell somewhere in-between that of the other two types of problems. Finally, analysis of the errors revealed a consistent pattern across conditions, with the selection of the prior probability value as the most common error.

## Study 3

Using different response elicitation methods (i.e., open-ended vs. multiple-choice questions), Studies 1 and 2 consistently found that the difficulty of a problem is influenced by its domain. Specifically, accuracy rate was lower in the medical than the abstract domain. However, aggregating the data from Studies 1 and 2 reveals a slightly different pattern of results: Although participants consistently performed worse on medical problems compared to those in the abstract domain within each value combination, their accuracy differed significantly only within the third value combination (see the analyses in the Online Supplementary Material (OSM)). These findings suggest that the differences between domains may be smaller than initially anticipated, which has implications for the sample size estimates used in Studies 1 and 2.

Furthermore, Studies 1 and 2 did not allow us to conclude if daily-life problems (i.e., non-medical problems with similar levels of background knowledge to medical problems) were more difficult than abstract ones or not, since participants’ performance on these problems fell in between those reported with medical and abstract problems without significantly differing from either.

In order to better explore the robustness and, possibly, the magnitude of the difference in accuracy rate between the medical, daily-life, and abstract domains, we conducted another study by involving a larger number of participants per condition and a smaller number of problems.

### Method

#### Participants

The minimum sample size needed for Study 3 was computed by performing an a priori power analysis using G*Power 3.1 (Faul et al., 2009), which indicated a minimum of 115 participants per condition needed to detect a small effect size of 0.15, assuming *α* = .05 and 1 – *β* = .95. The survey was kept active until at least 120 participants completed the task for each of the six conditions. Accordingly, we recruited a new sample of 729 UK residents (*M*_age_ = 39 years, *SD* = 12.9; 254 men, 474 women, one participant preferred not to declare their gender) using the Prolific platform. Most of them had an undergraduate (41.2%) or a graduate degree (18.1%), some had at least some college/university (24.8%) and the remaining participants completed up to high school diploma (15.9%). Participants received the same compensation as in Studies 1 and 2.

#### Materials and design

The pre-registered protocol of Study 3 can be found at https://osf.io/cfmvk. Study 3 employed a full between-subject design, in which the main independent variable was the domain of the problem (medical, daily-life, vs. abstract problem). We focused on a single type of evidence (i.e., testing) and value combinations 2 and 3 (see Table 1), for which participants showed the smallest and the greatest difference, respectively, between the medical and abstract domains in the aggregated analysis of Studies 1 and 2. Study 3 therefore employed six problems (3 domains × 2 value combinations).

As in Study 1, the dependent variable was the accuracy of participants’ responses to an open-ended probability question; responses that were not equivalent to the correct Bayesian answer were classified into the six error categories described above (i.e., “Sensitivity,” “Base rate only,” “Evidence only,” “Joint occurrence,” “Specificity,” and “Other”).

At the end of the task, participants were presented with the same multiple-choice question used in the previous two studies to check the believability of the probability values appearing in the medical and daily-life problems. In addition, as a further manipulation check, we added two new questions regarding the perceived severity and personal worry about the three medical conditions involved in the problems within the medical domain (i.e., osteoarthritis, Down syndrome, and celiac disease). In the severity question, participants were asked to rank the three medical conditions from the most to the least severe; in the personal worry question, they were asked to rank the same three conditions from the one that worried them the most to the one that worried them the least (for the exact wording, see the Appendix). Ranking judgments were used to prompt differentiation between these three medical conditions, the severity of which might be otherwise difficult to discriminate, and, as a consequence, limiting possible “ceiling effects.”

#### Results

Of the total sample, 8.8% of participants indicated that the numerical values provided in the problems were believable to them and aligned with their knowledge about the problem content; 63.8% indicated that the numerical values provided in the problems were believable to them but also that they had no knowledge about the specific content; 18.9% indicated that the values were at least partially believable to them and aligned with their knowledge about the specific content; and only 8.4% indicated that the values were unbelievable to them and not aligned with their knowledge. This distribution of answers did not differ significantly between medical and daily-life problems, nor between the two value combinations within the same domain (all *p*s > .05).

**Fig. 1:**
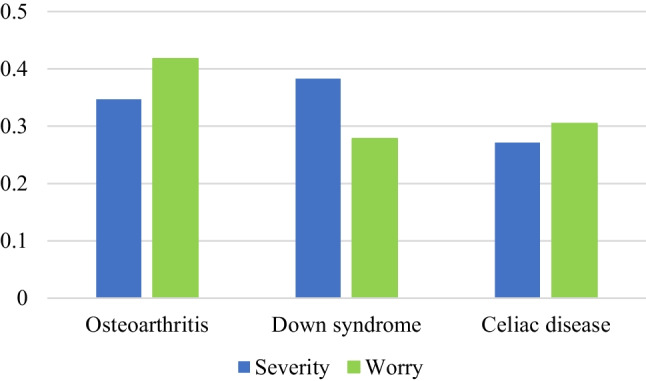
Participants’ rankings of severity and worry about the three medical conditions. To display participants’ assessments on a scale between 0 and 1, we assigned each medical condition a score from 1 (least severe/worrisome) to 3 (most severe/worrisome), and then normalized total scores using the MinMax normalization method

The results showed that, among participants who were presented with the medical problems, Down syndrome was ranked as the most severe of the three conditions, followed by osteoarthritis, and celiac disease as the least severe. However, participants expressed greater worry about osteoarthritis, followed by celiac disease, and the least worry about Down syndrome (see Fig. 1). These findings suggest that participants evaluated severity based on criteria other than personal worry, and their evaluations are particularly sensible when considering the age of our sample (M_age_ = 39 years). For example, having a child with Down syndrome, although considered a severe condition, may not worry the participants as much if they were already parents or were beyond their childbearing years. On the other hand, osteoarthritis, although considered comparatively less severe than Down syndrome, may worry them more because it is a medical condition that becomes more frequent with advancing age. Two separate logistic regression analyses were conducted to examine the relationship between participants’ accuracy and their rankings of severity and personal worry about the medical conditions (see the analyses in the OSM). One analysis was performed on participants who read the medical problems concerning Down syndrome (i.e., value combination 2), while the other analysis was conducted on those who read the medical problem concerning osteoarthritis (i.e., value combination 3). The results indicated that participants’ rankings of severity and personal worry did not predict the accuracy of their performance on either problem (all *p*s > .05).

Percentages of correct responses and distribution of non-Bayesian responses in the six conditions of Study 3 are reported in Tables 4 and 5, respectively. Yet again, participants’ accuracy rate was low (14.3%). However, unlike Studies 1 and 2, the proportion of Bayesian responses varied significantly among the six problems (*χ*^2^(5, *N* = 729) = 32.87, *p* < .001, *BF*_10_ > 10), indicating a clearer pattern of results. Indeed, the same logistic regression analysis performed in previous studies confirmed that participants were less accurate in the medical domain than in the abstract one (*OR* = .308, 95% CI, .182–.521; *p* < .001), but also show that they were less accurate in the daily-life domain than in the abstract one (*OR* = .300, 95% CI, .177–.507; *p* < .001), while, yet again, no difference was observed between the medical and the daily-life problems (all *p*s > .05). Consistent with Studies 1 and 2, the combination of values was not a significant predictor of participants’ accuracy rate (*p* > .05), but, importantly, the difference between domains was significant within each value combination (*χ*^2^(2, *N* = 364) = 13.53, *p* = .002, *BF*_10_ > 10, *χ*^2^(2, *N* = 365) = 19.01, *p* < .001, *BF*_10_ > 10 for value combinations 2 and 3, respectively).^5^

**Table 4:** Accuracy rates (i.e., percentages of Bayesian responses) for the six experimental conditions of Study 3. Refer to Table 1 for an explanation of values 2 and 3

Domain	Value combination	N	Accuracy %
Medical	2	120	9.2
	3	120	9.2
	Overall	240	9.2
Daily-life	2	123	8.9
	3	123	8.9
	Overall	246	8.9
Abstract	2	121	23.1
	3	122	26.2
	Overall	243	24.7
Total		729	14.3

**Table 5:** Percentages of non-Bayesian responses falling into the six incorrect categories in Study 3. Refer to Table 1 for an explanation of values 2 and 3

Domain	Value combination	N	Non-Bayesian responses					
			Sensitivity	Base rate	Evidence only	Joint occurrence	Specificity	Other
Medical	2	120	11.7	20.0	4.2	8.3	0.0	46.7
	3	120	14.2	10.0	0.0	10.8	0.0	55.8
	Overall	240	12.9	15.0	2.1	9.6	0.0	51.2
Daily-life	2	123	7.3	22.8	8.9	11.4	0.0	40.7
	3	123	6.5	12.2	24.4	11.4	0.0	36.6
	Overall	246	6.9	17.5	16.7	11.4	0.0	38.6
Abstract	2	121	12.4	15.7	13.2	4.1	0.0	31.4
	3	122	6.6	18.0	9.8	12.3	0.0	27.0
	Overall	143	9.5	16.9	11.5	8.2	0.0	29.2
Overall		729	9.7	16.5	10.2	9.7	0.0	39.6

Once again, the majority of participants’ non-Bayesian responses fell into the “Other” category (39.6% across all conditions). The most frequent error among the remaining response options was the “Base-rate only” category (16.5% across all conditions). Errors belonging to the “Evidence only,” “Joint occurrence,” and “Sensitivity” categories were approximately equally frequent (10.2%, 9.7%, and 9.7%, respectively), while no participants provided incorrect answers that fell into the “Specificity” category. No significant differences were observed in the distribution of errors among domains (*p* > .05).

## Discussion

We systematically investigated the impact of problem domain on Bayesian inference accuracy in three online studies, which involved 2,238 participants overall. To this aim, we employed isomorphic problems, that is, problems that were completely matched in terms of values and (as much as possible) wording, and varied only with regard to the domain. Our findings confirmed low accuracy rates for all the problems, in particular for those in the medical domain (Studies 1 and 2). Such a result, however, is only partially consistent with previous research, since Study 3 showed that the key difference in participants’ performance did not lie between medical and non-medical problems, as previously suggested. Rather, it appears to lie between abstract and real-life problems, whether the latter be in a medical or in a non-medical domain. Indeed, no difference was observed between medical and equally believable daily-life problems, while participants showed significantly higher accuracy rates when answering corresponding (in terms of values) abstract problems.

While providing a definitive explanation for participants’ different accuracy rates across the manipulated domains is beyond the scope of the present research, we are able to rule out at least some potential explanations with varying degrees of certainty. First, as all values in our studies were matched across domains, we can definitively exclude that the difficulty with medical problems is caused by the specific probability values employed in previous research (in particular the low base rates). Second, we can also reject the hypothesis of a main role of the type of evidence (i.e., the outcome of a test vs. a property probabilistically associated with the hypothesis under evaluation), since this variable was systematically manipulated across domains in the first two studies and did not appear to impact the accuracy rate. Third, at least regarding the medical problems, we can dismiss a main role of participants’ subjective feelings concerning the severity of the medical condition at issue or the subjective worry about it, as accuracy rate was unaffected by these evaluations. Fourth, it is unlikely that the effect of domain is due to difficulty understanding medical terminology, as our results showed that the accuracy in daily-life problems that did not employ technical terms (e.g., in the organic apples problem) was fully comparable to that of medical problems that used technical terms.

On the other hand, we cannot entirely exclude the possibility that participants’ implicit background knowledge about the content of the problem may have influenced their probabilistic inferences. In this regard, indeed, it is worth noting that the highest accuracy rates were observed in problems in the abstract domain, for which any potential interference from prior knowledge is, by definition, eliminated. The effects of realistic versus abstract domains on reasoning problems of various kinds is not a new finding in the psychology of thinking (e.g., Sperber, Cara, & Girotto, 1995), even if the direction of this effect is not always obvious (see, e.g., Wason, 1966, and Revlin et al., 1980). For example, arbitrary relations between symbols typically facilitate syllogistic reasoning by preventing the belief bias (Evans, Barston, & Pollard, 1983; Revlin et al., 1980) but are associated with a worse performance on the Wason selection task (Johnson-Laird, Legrenzi, & Legrenzi 1972; Girotto & Tentori, 2008). Future research may delve more deeply into these aspects, particularly investigating whether the advantage of abstract material in Bayesian word problems depends on a better comprehension or representation of the relevant contingencies. In this perspective, it might be helpful to connect our results with the substantial body of research that has examined the facilitatory role of graphical visualization methods (e.g., Brase, 2009, 2014; see Cui, Lo & Liu, 2023, for a review), in order to systematically explore the intersection between the abstractness of the domain and of the visual representations themselves and, possibly, to develop visualization aids for shifting individuals’ focus toward more abstract representations.

Notably, although the overall difference between domains was statistically significant, it appeared to be smaller than expected. All problems in this research were presented in a natural frequency format, which, according to the prevailing view, is considered to be the cognitively privileged representational format for Bayesian reasoning (e.g., Gigerenzer & Hoffrage, 1995, 2007; Hoffrage, Krauss, Martignon, & Gigerenzer, 2015). Consistent with previous online studies that employed the same numerical and question format (e.g., Micallef et al., 2012; Ottley et al., 2015; Pighin et al., 2016, 2018), the overall accuracy rate was low (17.2%, 17.4%, and 14.3% in Studies 1, 2 and 3, respectively). This does not support the mainstream stance (see Gigerenzer & Hoffrage, 1995; McDowell & Jacobs, 2017) that natural frequencies facilitate the solution of Bayesian word problems by a large number of individuals. In light of the above, we believe it is crucial to discuss two interconnected points. First, while the natural frequency format has repeatedly demonstrated a facilitatory effect over percentages (for a review, see McDowell & Jacobs, 2017), its actual benefits for the general population have often been overestimated (e.g., on this point, see also Garcia-Retamero & Hoffrage, 2013; Pighin et al. 2016; Siegrist & Keller, 2011). Indeed, even when framed in a natural frequency format, the Bayesian word problem poses an arduous challenge to resolve. This challenge, however, cannot be solely attributed to the difficulty of the computational calculations required, which are nearly eliminated using natural frequencies (Barbey & Sloman, 2007). The main difficulty, however, may reside in understanding the Bayesian word problem itself, which entails constructing and integrating an appropriate representation of all the conveyed information. Such a possibility remains speculative at the moment, and further studies are necessary to provide conclusive insights into this matter. Secondly, we hold the viewpoint that Bayesian word problems framed in a natural frequency format are a special instance of probabilistic updating problems. This becomes particularly evident when we consider that their correct solution can be obtained simply by dividing the number of true positives by the total number of positives. Given that in the natural frequency format subsets inherently integrate the base-rate information, individuals can overlook the specific value about the latter, which is typically provided at the beginning of the problem. Nevertheless, the most common error with this format is to precisely report such a value. This pattern is exactly the opposite of what the existing literature (and label) on base-rate neglect has suggested over the past 40 years: a tendency to disregard or underweight priors in probabilistic updating. Thus, we acknowledge that the results of this study cannot be directly extrapolated to problems in which information is presented in other numerical formats (such as percentages), in which different errors are commonly observed. At the same time, we are aware that other numerical formats would easily lead to a reduction in accuracy rates and, then, create a significant methodological challenge by further obscuring any potential domain effect.

To conclude, our findings provide a methodological guidance for investigating Bayesian inference through word problems, promoting greater awareness of the potential impact that the specific domains employed may have on participants’ accuracy rate. They also offer new insights into the ambitious challenge of improving Bayesian inference, highlighting the need for further investigation into overcoming a specific difficulty associated with real-life domains, such as the medical one, where Bayesian reasoning has its important applications.

## Data Availability

Complete datasets and supplementary analyses can be found at the following repository: https://osf.io/p37nz/

## Appendix

The Bayesian word problems used in Study 2 are detailed below. In Studies 1 and 3, the problems were identical, except that response options were not provided, and participants were asked to answer by filling in the numerator and denominator in the sentence “____ out of _____”. Please note that in Study 3, we used only six of the 18 problems listed below, omitting the first six problems (i.e., those with value combination 1) as well as the problems in the non-testing versions. The information presented in parentheses was not shown to the participants.

Exact wording of the severity and worry questions employed in Study 3. The presentation order of the three medical conditions was randomized across participants.

## Footnotes

1 The four response options were formulated with the aim of maintaining task brevity and manageability, under the assumption that participants’ evaluation of believability depends on their knowledge of the subject. From this standpoint, only a limited number of options appeared to make sense to us, given that, for example, participants are unlikely to regard values as believable if these are not (even partially) aligned with their knowledge. Symmetrically, we expected that participants would have no reason to find values only partially believable or unbelievable if they completely lack knowledge of the specific topic. However, different response options could have been formulated, or two separate questions (one for believability and one for knowledge) could have been employed (we thank Dr. Lucy Cui, one of the reviewers of this paper, for the latter suggestion). Note, however, that the specific question used is unlikely to have affected the interpretation of results, given the lack of correlation between higher believability and background knowledge on the one hand and accuracy on the other, reported in Study 2.

2 *BF*_10_ quantifies the evidence in support of the alternative hypothesis over the null hypothesis; *BF*_10_ values are typically interpreted according to the following convention: >10 = “strong evidence for H_1_”; 10–3 = “moderate evidence for H_1_”; “3–1” = “anecdotal evidence for H_1_”; “1” = “equal evidence for H_1_ and H_0_”; 1–1/3 = “anecdotal evidence for H_0_”; 1/3–1/10 = “moderate evidence for H_0_”; < 1/10 = “strong evidence for H_0_” (e.g., Lee & Wagenmakers, 2014).

3 In all three studies, the logistic regression analyses and the analyses involving manipulation checks were exploratory and not pre-registered.

4 A particularly bad performance was observed in the medical scenario with the third set of values and non-testing evidence. We lack a definitive explanation for this specific result, even if we can confidently rule out that it was due to the specific values used, as they were matched across scenarios. It’s possible that unfamiliarity with the two conditions (osteoarthritis and osteoporosis) and their potential causal links played a role, even if the accuracy rate was similar to that of other medical conditions in our study. Another possibility is confusion between “osteo-” prefixes, derived from Latin “os” meaning “bone,” which could have affected identification of relevant subsets. However, this remains speculative, and the result may well be due to random noise.

5 Reported *p*-values were adjusted with Bonferroni correction for multiple comparisons.

## References

[CR1] Barbey AK, Sloman SA (2007). Base-rate respect: From ecological rationality to dual processes. Behavioral and Brain Sciences.

[CR2] Bar-Hillel M (1980). The Base-Rate Fallacy in Probability Judgments. Acta Psychologica.

[CR3] Binder K, Krauss S, Bruckmaier G (2015). Effects of visualizing statistical information–an empirical study on tree diagrams and 2× 2 tables. Frontiers in Psychology.

[CR4] Brase GL (2009). Pictorial representations in statistical reasoning. Applied Cognitive Psychology.

[CR5] Brase GL (2014). The power of representation and interpretation: doubling statistical reasoning performance with icons and frequentist interpretations of ambiguous numbers. Journal of Cognitive Psychology.

[CR6] Bruckmaier G, Binder K, Krauss S, Kufner HM (2019). An eye-tracking study of statistical reasoning with tree diagrams and 2× 2 tables. Frontiers in Psychology.

[CR7] Chapman GB, Liu J (2009). Numeracy, frequency, and Bayesian reasoning. Judgment and Decision Making.

[CR8] Chater N, Oaksford M (2008). *The probabilistic mind: Prospects for Bayesian cognitive science*.

[CR9] Cui L, Lo S, Liu Z (2023). The Use of Visualizations to Improve Bayesian Reasoning: A Literature Review. Vision.

[CR10] Ellsberg D (1961). Risk, ambiguity, and the Savage axioms. The Quarterly Journal of Economics.

[CR11] Evans JSB, Barston JL, Pollard P (1983). On the conflict between logic and belief in syllogistic reasoning. Memory & Cognition.

[CR12] Faul F, Erdfelder E, Buchner A, Lang AG (2009). Statistical power analyses using G* Power 3.1: Tests for correlation and regression analyses. Behavior Research Methods.

[CR13] Garcia-Retamero R, Hoffrage U (2013). Visual representation of statistical information improves diagnostic inferences in doctors and their patients. Social Science & Medicine.

[CR14] Gigerenzer G, Hoffrage U (1995). How to improve Bayesian reasoning without instruction: Frequency formats. Psychological Review.

[CR15] Gigerenzer G, Hoffrage U (2007). The role of representation in Bayesian reasoning: correcting common misconceptions. Behavioral and Brain Sciences.

[CR16] Girotto V, Tentori K (2008). Is domain-general thinking a domain-specific adaptation?. Mind & Society.

[CR17] Hafenbrädl S, Hoffrage U (2015). Toward an ecological analysis of Bayesian inferences: how task characteristics influence responses. Frontiers in Psychology.

[CR18] Hammerton M (1973). A case of radical probability estimation. Journal of Experimental Psychology.

[CR19] Heath C, Tversky A (1991). Preference and belief: Ambiguity and competence in choice under uncertainty. Journal of Risk and Uncertainty.

[CR20] Hoffrage U, Krauss S, Martignon L, Gigerenzer G (2015). Natural frequencies improve Bayesian reasoning in simple and complex inference tasks. Frontiers in Psychology.

[CR21] Johnson ED, Tubau E (2015). Comprehension and computation in Bayesian problem solving. Frontiers in Psychology.

[CR22] Johnson-Laird PN, Legrenzi P, Legrenzi MS (1972). Reasoning and a sense of reality. British Journal of Psychology.

[CR23] Kahneman D, Tversky A (1973). On the psychology of prediction. Psychological Review.

[CR24] Lee MD, Wagenmakers EJ (2014). *Bayesian cognitive modeling: A practical course*.

[CR25] Lyman GH, Balducci L (1993). Overestimation of test effects in clinical judgment. Journal of Cancer Education.

[CR26] McDowell M, Jacobs P (2017). Meta-analysis of the effect of natural frequencies on Bayesian reasoning. Psychological Bulletin.

[CR27] McNair S, Feeney A (2014). When does information about causal structure improve statistical reasoning?. Quarterly Journal of Experimental Psychology.

[CR28] Micallef L, Dragicevic P, Fekete JD (2012). Assessing the effect of visualizations on bayesian reasoning through crowdsourcing. IEEE Transactions on Visualization and Computer Graphics.

[CR29] Msaouel P, Kappos T, Tasoulis A, Apostolopoulos AP, Lekkas I, Tripodaki ES, Keramaris NC (2015). Comparison of resident performance in interpreting mammography results using a probabilistic or a natural frequency presentation: A multi-institutional randomized experimental study. Education for Health.

[CR30] Ottley A, Peck EM, Harrison LT, Afergan D, Ziemkiewicz C, Taylor HA, Chang R (2015). Improving Bayesian reasoning: The effects of phrasing, visualization, and spatial ability. IEEE tTransactions on Visualization and Computer Graphics.

[CR31] Pighin, S., Girotto, V., & Tentori, K. (2017). Children’s quantitative Bayesian inferences from natural frequencies and number of chances. *Cognition, 168*, 164–175. 10.1016/j.cognition.2017.06.028 28692831

[CR32] Pighin S, Gonzalez M, Savadori L, Girotto V (2016). Natural frequencies do not foster public understanding of medical test results. Medical Decision Making.

[CR33] Pighin S, Tentori K (2021). Public’s understanding of swab test results for SARS-CoV-2: an online behavioural experiment during the April 2020 lockdown. BMJ open.

[CR34] Pighin, S., Tentori, K., & Girotto, V. (2017). Another chance for good reasoning. *Psychonomic Bulletin & Review, 24*, 1995–2002. 10.3758/s13423-017-1252-5 28265865

[CR35] Pighin S, Tentori K, Savadori L, Girotto V (2018). Fostering the understanding of positive test results. Annals of Behavioral Medicine.

[CR36] Revlin R, Leirer V, Yopp H, Yopp R (1980). The belief-bias effect in formal reasoning: The influence of knowledge on logic. Memory & Cognition.

[CR37] Siegrist M, Keller C (2011). Natural frequencies and Bayesian reasoning: the impact of formal education and problem context. Journal of Risk Research.

[CR38] Sirota M, Juanchich M, Hagmayer Y (2014). Ecological rationality or nested sets? Individual differences in cognitive processing predict Bayesian reasoning. Psychonomic Bulletin & Review.

[CR39] Sperber D, Cara F, Girotto V (1995). Relevance theory explains the selection task. Cognition.

[CR40] Steurer J, Fischer JE, Bachmann LM, Koller M, ter Riet G (2002). Communicating accuracy of tests to general practitioners: a controlled study. Bmj.

[CR41] Sutton, R. S., & Barto, A. G. (2018). *Reinforcement learning: An introduction*. MIT press.

[CR42] Tentori K, Crupi V, Osherson D (2007). Determinants of confirmation. Psychonomic Bulletin and Review.

[CR43] Tversky A, Kahneman D (1974). Judgment under Uncertainty: Heuristics and Biases: Biases in judgments reveal some heuristics of thinking under uncertainty. Science.

[CR44] Wason, P. C. (1966). *Reasoning*. In B. Foss (Ed.), New horizons in psychology (pp. 135–151). Harmondsworth, Middlesex, England: Penguin.

